# Comparative Genomics of Community-Acquired ST59 Methicillin-Resistant *Staphylococcus aureus* in Taiwan: Novel Mobile Resistance Structures with IS*1216V*

**DOI:** 10.1371/journal.pone.0046987

**Published:** 2012-10-05

**Authors:** Wei-Chun Hung, Tomomi Takano, Wataru Higuchi, Yasuhisa Iwao, Olga Khokhlova, Lee-Jene Teng, Tatsuo Yamamoto

**Affiliations:** 1 Division of Bacteriology, Department of Infectious Disease Control and International Medicine, Niigata University Graduate School of Medical and Dental Sciences, Niigata, Japan; 2 Department of Clinical Laboratory Sciences and Medical Biotechnology, National Taiwan University College of Medicine, Taipei, Taiwan; National Institutes of Health, United States of America

## Abstract

Methicillin-resistant *Staphylococcus aureus* (MRSA) with ST59/SCC*mec*V and Panton-Valentine leukocidin gene is a major community-acquired MRSA (CA-MRSA) lineage in Taiwan and has been multidrug-resistant since its initial isolation. In this study, we studied the acquisition mechanism of multidrug resistance in an ST59 CA-MRSA strain (PM1) by comparative genomics. PM1’s non-β-lactam resistance was encoded by two unique genetic traits. One was a 21,832-bp composite mobile element structure (MES_PM1_), which was flanked by direct repeats of enterococcal IS*1216V* and was inserted into the chromosomal *sasK* gene; the target sequence (*att*) was 8 bp long and was duplicated at both ends of MES_PM1_. MES_PM1_ consisted of two regions: the 5′-end side 12.4-kb region carrying Tn*551* (with *ermB*) and Tn*5405*-like (with *aph[3′]-IIIa* and *aadE*), similar to an *Enterococcus faecalis* plasmid, and the 3′-end side 6,587-bp region (MES*_cat_*) that carries *cat* and is flanked by inverted repeats of IS*1216V*. MES*_cat_* possessed *att* duplication at both ends and additional two copies of IS*1216V* inside. MES_PM1_ represents the first enterococcal IS*1216V*-mediated composite transposon emerged in MRSA. IS*1216V*-mediated deletion likely occurred in IS*1216V*-rich MES_PM1_, resulting in distinct resistance patterns in PM1-derivative strains. Another structure was a 6,025-bp *tet*-carrying element (MES*_tet_*) on a 25,961-bp novel mosaic penicillinase plasmid (pPM1); MES*_tet_* was flanked by direct repeats of IS*431*, but with no target sequence repeats. Moreover, the PM1 genome was deficient in a copy of the restriction and modification genes (*hsdM* and *hsdS*), which might have contributed to the acquisition of enterococcal multidrug resistance.

## Introduction

Methicillin-resistant *Staphylococcus aureus* (MRSA) is a major public health problem worldwide. It was first isolated in 1961 as a nosocomial pathogen [1], [2], now known as hospital-acquired MRSA (HA-MRSA). Another class of MRSA, designated community-acquired MRSA (CA-MRSA), emerged in the community from 1997 to 1999, posing a novel threat worldwide [1]–[4]. CA-MRSA infection usually occurs in children and young adults, or even the elderly, without exposure to hospital environments, and includes common skin and soft tissue infections (SSTIs) and occasionally life-threatening invasive infections, such as sepsis, necrotizing pneumonia and osteomyelitis [1]–[5].

CA-MRSA is characterized by the presence of staphylococcal cassette chromosome *mec* type IV (SCC*mec*IV) or SCC*mec*V [1], [2], [4], [6]. SCC*mec*IV likely contributes to the spread in the community because it is smaller in size compared to other SCC*mec* elements [4], [6], [7] and has no effect on MRSA fitness (engendering no biological cost) [4], [8]. CA-MRSA strains often produce Panton-Valentine leukocidin (PVL), which acts against neutrophils [4], [9]–[11]. Moreover, CA-MRSA strains are only resistant to β-lactam antimicrobial agents or to some agents in restricted classes, although some recently successful CA-MRSA strains, such as multilocus sequence type 8 (ST8) CA-MRSA USA300, became multidrug-resistant [12], [13]; USA300 has even spread into hospitals as a nosocomial pathogen [14], [15].

A major CA-MRSA lineage in Taiwan is ST59 with SCC*mec*V and *luk_PV_SF* genes encoding PVL. Wang et al. [16] and Boyle-Vavra et al. [17] demonstrated that ST59 CA-MRSA in Taiwan was already resistant to multidrugs (including erythromycin, clindamycin, and chloramphenicol) when it began emerging in 1997, in contrast to the general understanding of CA-MRSA. Boyle-Vavra et al. [17] also characterized the SCC*mec* of Taiwanese ST59 CA-MRSA (representative strain, TSGH17) as SCC*mec* V_T_. The ST59 CA-MRSA lineage (with SCC*mec* type V_T_ and *luk_PV_SF*) has also been reported in Singapore [18], Hong Kong [19], Japan [20], [21], and Western Australia [22].

We further characterized the Taiwanese ST59 CA-MRSA lineage and found that it possessed a novel SCC*mec* with two dictinct *ccrC* genes (*ccrC1* allele 2 and *ccrC1* allele 8) [23]–[25]. We determined the entire SCC*mec* sequence of the ST59 CA-MRSA lineage (strain PM1) and tentatively designated it as SCC*mec*VII [24]; later, the SCC*mec* of the Taiwanese ST59 CA-MRSA lineage (strains TSGH17 and PM1) was reclassified as SCC*mec*V by the International Working Group on the Classification of Staphylococcal Cassette Chromosome Elements [6]. As for non-β-lactam resistance, three resistance determinants, *ermB* (encoding erythromycin/clindamycin resistance), *aph(3′)-IIIa* (encoding kanamycin resistance), and *aadE* (encoding streptomycin resistance), were clustered in a region, previously named the drug resistance gene cluster (*rgc*) [23].

IS*1216V* is an enterococcal insertion sequence [26] and is rarely found in *S. aureus*. IS*1216V*-positive *S. aureus* cases include vancomycin-resistant MRSA (VRSA), in which IS*1216V* is inserted in a vancomycin resistance transposon (Tn*1546*) [27]. The *rgc* region of strain PM1 also carried IS*1216V* [23], raising the question of whether the *rgc* region of the Taiwanese ST59 CA-MRSA lineage (strain PM1) originated in enterococci through an IS*1216V* function.

In order to further understand the mechanism of multidrug resistance acquisition by PVL-positive ST59 CA-MRSA in Taiwan, we performed comparative genomics of strain PM1 and found that strain PM1 became multidrug resistant by acquiring two mobile genetic elements, an enterococcal IS*1216V*-mediated composite mobile element structure (MES_PM1_) on the chromosome and an IS*431*-mediated element (MES*_tet_*) on a plasmid, besides β-lactam resistance by SCC*mec*V. We also searched for the genetic background of the ST59 CA-MRSA lineage, which allowed (or stimulated) the acquisition of multidrug resistance.

## Results

### Susceptibility to Non-β-lactam Agents of PVL-positive ST59/SCC*mec*V CA-MRSA Strains in Taiwan

Drug resistance patterns of ST59/SCC*mec*V CA-MRSA strains isolated in Taiwan, compared to an ST59 MRSA-type strain USA1000, are summarized in Table 1. Half of the strains (including PM1) were resistant to erythromycin/clindamycin, kanamycin, streptomycin, chloramphenicol, and tetracycline, in addition to β-lactam agents, showing marked multidrug resistance (in contrast to USA1000). The rest showed five distinct patterns in terms of resistance to non-β-lactam agents: resistance to erythromycin/clindamycin, kanamycin, streptomycin, and chloramphenicol; resistance to erythromycin/clindamycin, kanamycin, streptomycin, and tetracycline; resistance to erythromycin/clindamycin, kanamycin, and streptomycin; resistance to chloramphenicol and tetracycline; and non-resistance (to any non-β-lactam agent).

**Table 1:** Resistance patterns of ST59 CA-MRSA strains isolated in Taiwan, compared to ST59 MRSA USA1000.

Resistance patterns^a^					Percentage (no./total)	Example (strain)	Plasmid^b^ (size)
Ery/Cli	Kan	Stm	Chl	Tet			
R	R	R	R	R	50% (12/24)	PM1, PM22	26 kb
R	R	R	R	S	16.7% (4/24)	PM11	21 kb
R	R	R	S	R	16.7% (4/24)	PM9	26 kb
R	R	R	S	S	8.3% (2/24)	PM12	None
S	S	S	R	R	4.2% (1/24)	PM8	26 kb
S	S	S	S	S	4.2% (1/24)	PM18	21 kb
R(Ery)	S	S	S	S	-	USA1000	ND^c^

a The number of strains tested was 24. Non-β-lactam agents: Ery, erythromycin; Cli, clindamycin; Kan, kanamycin; Stm, streptomycin; Chl, chloramphenicol; Tet, tetracycline. USA1000 is a type strain of PVL-positive ST59 MRSA.

b Plasmid analysis was carried out for example strains.

c Not determined.

### Comparative Genomics of PVL-positive Multidrug-resistant ST59/SCC*mec*V CA-MRSA (Strain PM1)

**Figure 1:**
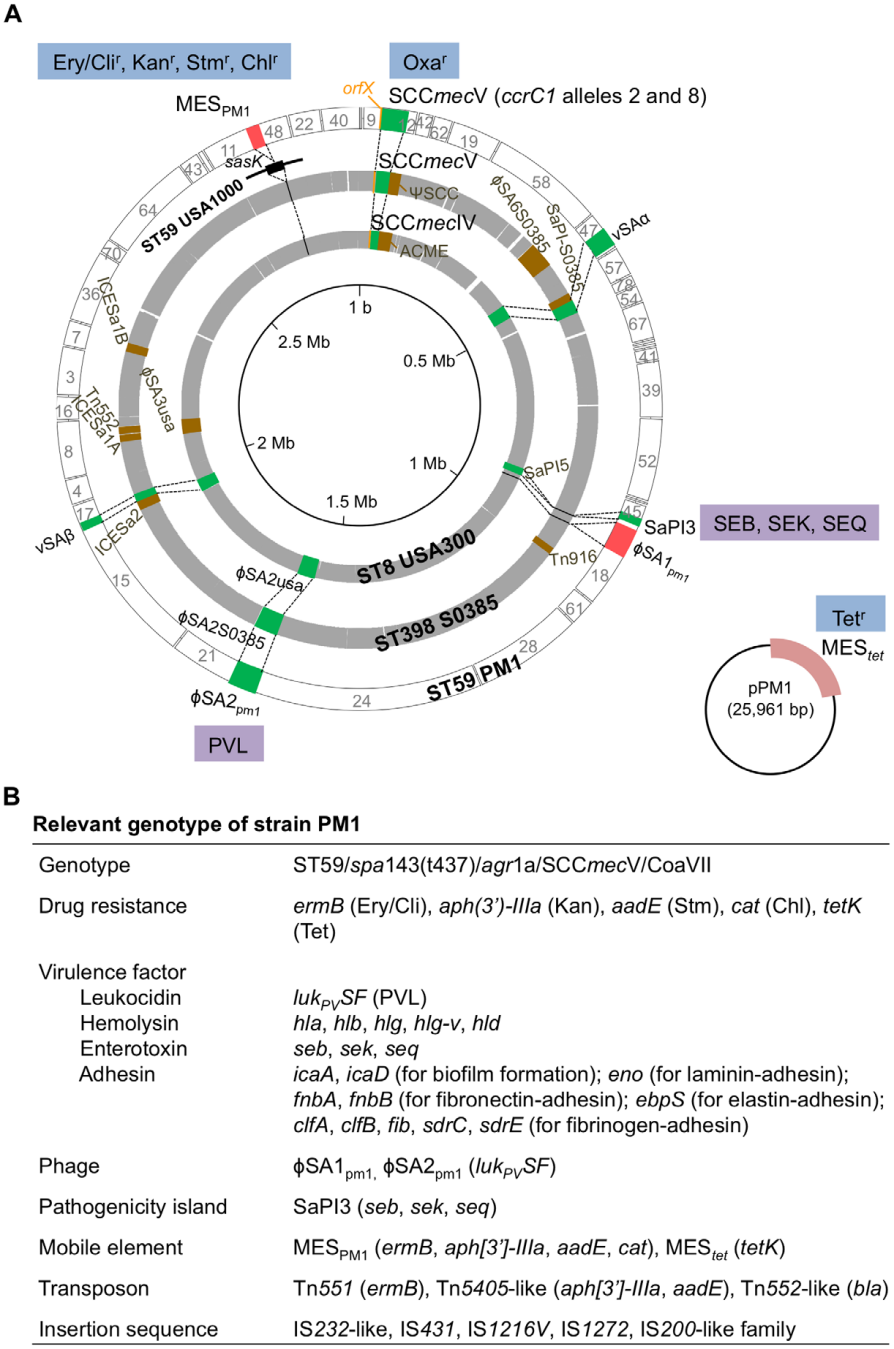
Genome comparisons of ST59 MRSA strain PM1 with two other *S. aureus* genomes. In A, two gray circles show the complete genomes of ST8 CA-MRSA strain USA300 FPR3757 (2,872,769 bp) and ST398 MRSA strain S0385 (2,872,582 bp); gaps in the genome circles indicate USA300 or S0385 sequences not present in PM1. The *sasK* gene with the MES_PM1_ integration site (*att*) is shown outside the gray circles (on the USA1000 genome circle), since the USA300 and S0385 MRSA strains do not possess the *sasK* gene. The outermost (white) circle represents the alignment of PM1 contigs and provides PM1 genome information, including drug resistance traits, pathogenetic islands, bacteriophages, and genomic islands νSAα and νSAβ. Plasmid pPM1 is shown in the right bottom of the figure. Color region in the genome: orange, *orfX* with the *att* sequence for SCC*mec*; green, genetic elements with diversity; brown, genetic elements present in USA300 or S0385 (but not in PM1); pink, genetic elements present in PM1 (but not in USA300 or S0385). In B, relevant genotypes of PM1 is summarized. Drug resistance (r): Ery/Cli, erythromycin/clindamycin; Kan, kanamycin; Stm, streptomycin; Chl, chloramphenicol; Oxa, oxacillin; Tet, tetracycline. Virulence factors: SEB, staphylococcal enterotoxin B; SEK, staphylococcal enterotoxin K; SEQ, staphylococcal enterotoxin Q; PVL, Panton-Valentine leukocidin.

The PM1 genome was analyzed by pyrosequencing (GenBank Accession number BAFA01000000) and compared with the genomes of ST398 MRSA human strain S0385 and PVL-positive ST8 CA-MRSA USA300, since ST398 MRSA carried SCC*mec*V [28], similar to PM1, and showed the highest similarity to ST59 MRSA [29] in terms of the seven housekeeping gene sequences used for multilocus sequence typing, and USA300 was one of the best characterized CA-MRSA [30]. The data are summarized in Figure 1. The alignment of PM1 contigs on the reference MRSA genome was the same in both cases (Figure 1A); the similarities of the PM1 genome to the ST398 and ST8 genomes were both approximately 98%, albeit with divergence in, for example, the carriage of mobile elements (Figure 1A). The PM1 genome was estimated to be at least 2.8 Mb. Strain PM1 carried a 25,961-bp plasmid (pPM1), encoding tetracycline resistance; plasmid DNA analysis of strain PM1 also demonstrated a single species of plasmid of 26.0 kb (Table 1).

When PM1 was compared with S0385 and USA300, the four regions were unique to PM1: i) SCC*mec*V with two *ccrC* genes (*ccrC1* allele 2 and *ccrC1* allele 8); ii) φSA1_PM1_, which was 70.2% homologous to φETA3, a member of the φSA1 family (φETA3 is *eta*-positive, while φSA1_PM1_ was negative); iii) MES_PM1_, a novel mobile element structure on the chromosome, encoding multidrug resistance; and iv) a tetracycline resistance plasmid pPM1. Moreover, νSAβ on the PM1 genome had a large deletion.

### Structure of MES_PM1_

**Figure 2:**
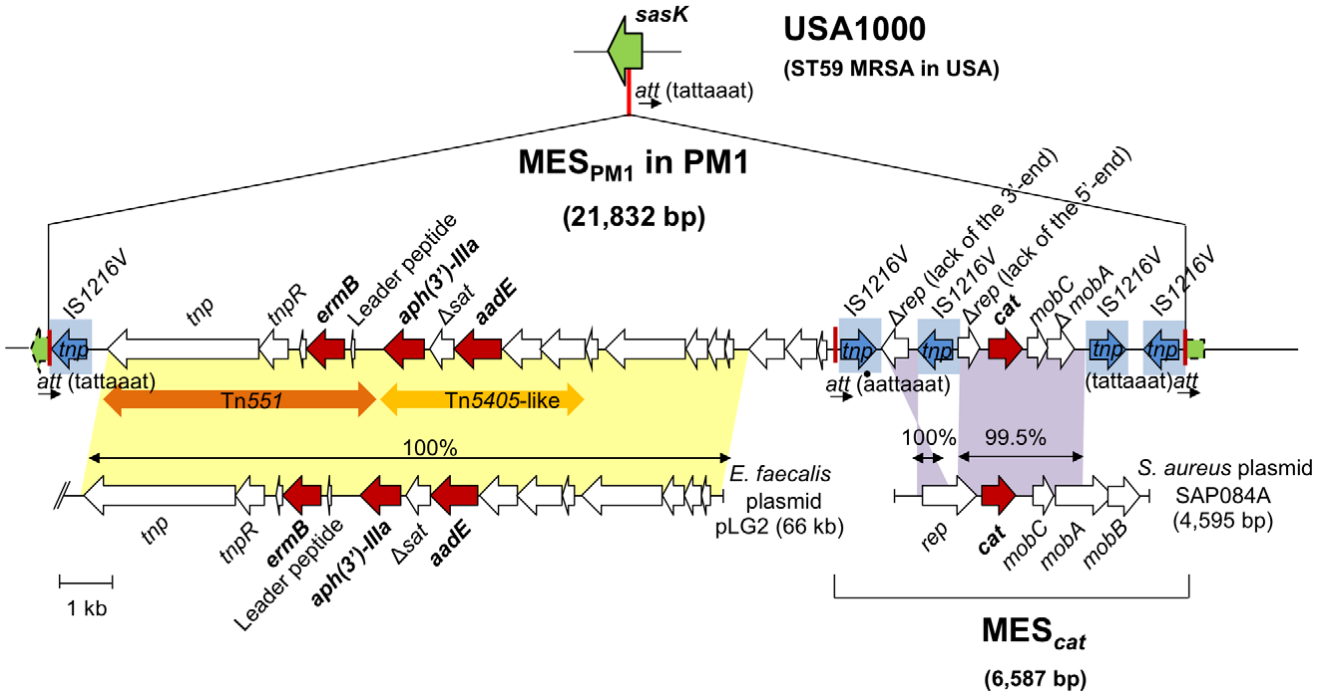
Genetic structure of MES_PM1_ carrying four resistance genes, *ermB*, *aph(3′)-IIIa*, *aadE*, and *cat*. MES_PM1_ was integrated into and disrupted the *sasK* gene, which encoded a putative cell-wall anchored surface protein with the LPXTG-motif. The intact *sasK* gene was present in PVL-positive ST59 CA-MRSA type strain USA1000. Three *att* sites are in a vertical red line. The region of IS*1216V* with the transposase gene (*tnp*) is shaded in blue. Homology regions with *E. faecalis* pLG2 plasmid (GenBank accession number HQ426665) and *S. aureus* SAP084A (GenBank accession number GQ900436) are shaded in yellow and purple, respectively. A Greek delta symbol indicates truncated coding sequence.

The entire sequence of MES_PM1_ and its surrounding region was determined (GenBank Accession number AB699882); the structure is summarized in Figure 2. MES_PM1_ was 21,832 bp long and flanked by direct repeats of IS*1216V* at both ends. It was inserted into the *att* site (8-bp target sequence) within the *sasK* gene; the 8-bp *att* sequence was duplicated at both ends of MES_PM1_, indicating that MES_PM1_ is a large transposon. The target gene (*sasK*) with the 8-bp *att* sequence was present on the genome of ST59 MRSA USA1000, but not in strains S0385 and USA300 (Figure 1A). Interestingly, MES_PM1_ was IS*1216V*-rich and contained three additional copies of IS*1216V* (a total of five copies of IS*1216V*), as shown Figure 2.

The left side of MES_PM1_ was a composite transposon region consisting of Tn*551* carrying *ermB* and Tn*5405*-like carrying *aph(3′)-IIIa* and *aadE*. The composite transposon region was identical to the corresponding region of a plasmid (pLG2) of *Enterococcus faecalis* (Figure 2); the entire sequence of pLG2 has not been reported.

Analysis of the right-side region of MES_PM1_ revealed an independent transposon (MES*_cat_*) carrying *cat* (encoding chloramphenicol resistance). MES*_cat_* was 6,587 bp long and flanked by the inverted repeats of IS*1216V*; moreover, the 8-bp *att* sequence was duplicated at both ends of MES*_cat_* (Figure 2). MES*_cat_* contained two additional copies of IS*1216V*. The central *cat* region in MES*_cat_* was highly homologous (99.5%) to the *cat* region of SAP084A plasmid in *S. aureus*.

Based on the data, we conclude that MES_PM1_ is a highly composite transposon, which originated in enterococci, but acquired an additional drug resistance gene (*cat*) in *S. aureus*. Another multidrug-resistant strain PM22 (Table 1) also possessed an MES structure (MES_PM22_) very similar to MES_PM1_, although there was a small deletion within the structure (data not shown).

### Structure of MES_PM1_ Segregants with Different Resistance Patterns

**Figure 3:**
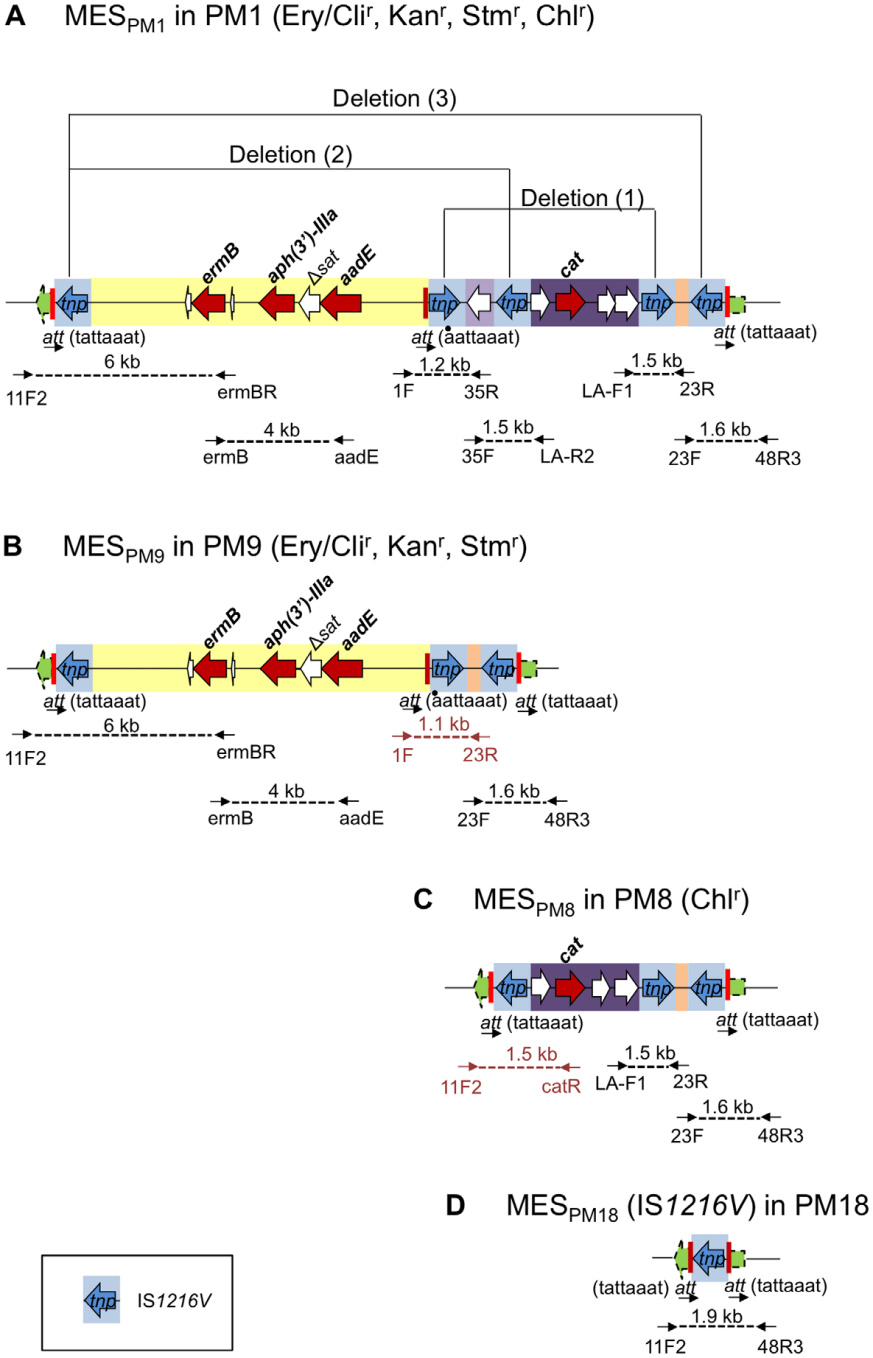
The structure of MES_PM1_ and its segregants in ST59 CA-MRSA strains. Structures of MES_PM1_, MES_PM9_, MES_PM8_, and MES_PM18_ (corresponding to IS*1216V*) are shown in A to D. Drug resistance (r): Ery/Cli, erythromycin/clindamycin; Kan, kanamycin; Stm, streptomycin; Chl, chloramphenicol. The IS*1216V* region with the transposase gene (*tnp*) is shaded in blue. The *att* site is indicated by a vertical red line. Arrows below the MES structures indicate PCR primers, which are listed in Materials and Methods.

The MES_PM1_-corresponding region of ST59 CA-MRSA strains with different resistance patterns (Table 1) was determined by sequencing (Figure 3). MES_PM1_ seems genetically unstable, and recombination likely takes place between two direct repeats of IS*1216V*, resulting in deletion, which could explain the distinct resistance patterns of ST59 CA-MRSA.

For instance, strain PM9, which was resistant to erythromycin/clindamycin, kanamycin, and streptomycin, but susceptible to chloramphenicol, seemed to be a segregant, generated through deletion (1) in Figure 3 (A, B). Likewise, strain PM8, which was resistant to chloramphenicol only, seemed to be a segregant, generated through deletion (2) in Figure 3 (A, C). Strain PM18, which was susceptible to all the non-β-lactam agents, seemed to be a segregant, generated through deletion (3) in Figure 3 (A, D).

MES_PM1_ segregants, MES_PM9_ (Figure 3B) and MES_PM8_ (Figure 3C), may also behave as transposons, since they were flanked by direct repeats of IS*1216V* at both ends with *att* sequence duplication and inserted into the *sasK* gene, just like MES_PM1_. MES_PM18_ (corresponding to IS*1216V*), which was inserted into the *sasK* gene and possessed *att* sequence duplication at both ends (Figure 3D), may behave as an IS.

### Structure of pPM1

**Figure 4:**
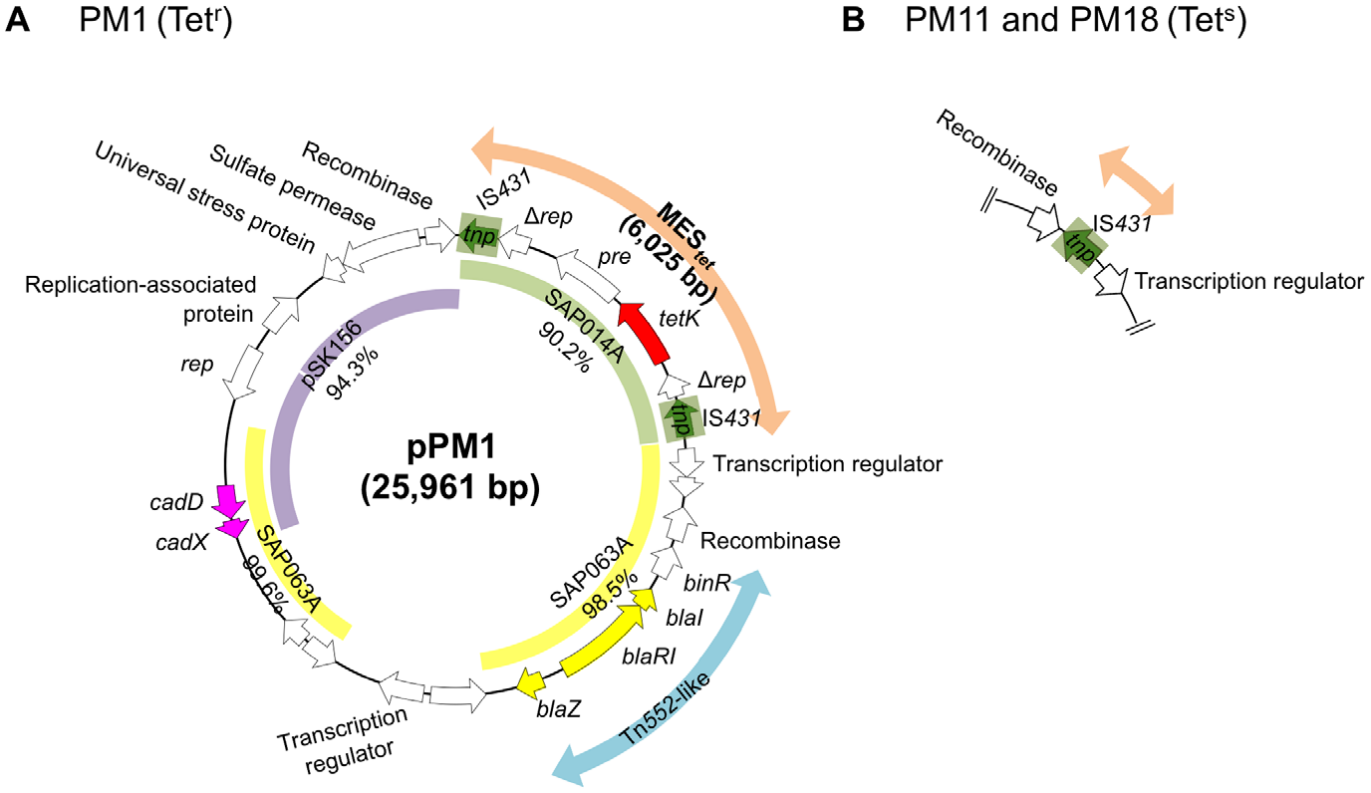
Genetic structure of penicillinase plasmid pPM1 and its segregant. In A, pPM1 structure is shown. Inner circle indicates homologous regions to *S. aureus* plasmid SAP014A (GenBank accession number GQ900379), SAP063A (GenBank accession number GQ900418), and pSK156 (GenBank accession number GQ900448). The IS*431* region with the transposase gene (*tnp*) is shaded in light green. A Greek delta symbol indicates truncated coding sequence. In B, a segregant of pPM1 in MES*_tet_* locus is shown. Tet^r^, tetracycline-resistant; Tet^s^, tetracycline-susceptible.

The entire sequence of pPM1 was determined (GenBank Accession number AB699881) and its structure is summarized in Figure 4A. pPM1 was a novel composite plasmid, consisting of a replication (*rep*) region (homologous to pSK156 plasmid), cadmium resistance (*cad*) region (homologous to SAP063A plasmid), ampicillin resistance (*bla*) region (homologous to SAP063A plasmid), and tetracycline resistance (*tetK*) region (homologous to SAP014A plasmid). The *tetK*-carrying region was 6,025 bp long and was flanked by direct repeats of IS*431* at both ends; this structure was designated MES*_tet_*. There was no *att* sequence duplication at either end of MES*_tet_*.

### Analysis of Tetracycline-susceptible Strains

Of three tetracycline-susceptible strains (Table 1) examined, two strains, PM11 and PM18, carried a pPM1-related plasmid (pPM11 and pPM18, respectively). Entire sequence analysis revealed that the two plasmids carried one copy of IS*431* only at the MES*_tet_* insertion site on pPM1, as shown in Figure 4B; therefore, pPM11 and pPM18 could be a segregant of pPM1 generated through a recombination/deletion event between two direct repeats of IS*431*. The remaining strain (PM12) lacked pPM1 plasmid.

### Conjugative Transfer of Drug Resistance

Conjugative transfer of drug resistance was performed between PM1 (donor strain) and *S. aureus* RN2677 (recipient strain) by filter mating. As shown in Table 2, resistance to ampicillin, tetracycline, and cadmium was transferred to *S. aureus* RN2677 with equal frequency; all transconjugants obtained carried a 26-kp penicillinase plasmid (pPM1), as expected. However, no MES_PM1_-associated transconjugants (exhibiting resistance to erythromycin, clindamycin, chloramphenicol or streptomycin) were obtained.

**Table 2:** Transfer of drug resistance in mating between ST59 CA-MRSA strain PM1and *S. aureus* RN2677.

Selection of transconjugant^a^	Transfer frequency: drug-resistant recipient/donor	Minimal inhibitory concentration (µg/ml)		
		Transconjugants	Recipient (RN2677)	Donor (PM1)
Ampicillin	1.6×10^−7^	1^b^	0.13	32
Tetracycline	1.6×10^−7^	32	0.25	32
Cadmium	1.6×10^−7^	32	4	32
Erythromycin	<1.4×10^−9^	–c		
Clindamycin	<1.4×10^−9^	–c		
Chloramphenicol	<1.0×10^−8^	–c		
Streptomycin	<1.0×10^−8^	–c		

a Novobiocin was used for selection of RN2677.

b MIC of ampicillin for transconjugants was lower than that for PM1 (1 µg/ml vs. 32 µg/ml).

c No transconjugants were obtained.

### Restriction and Modification Genes (*hsdS* and *hsdM*) in Strain PM1

**Figure 5:**
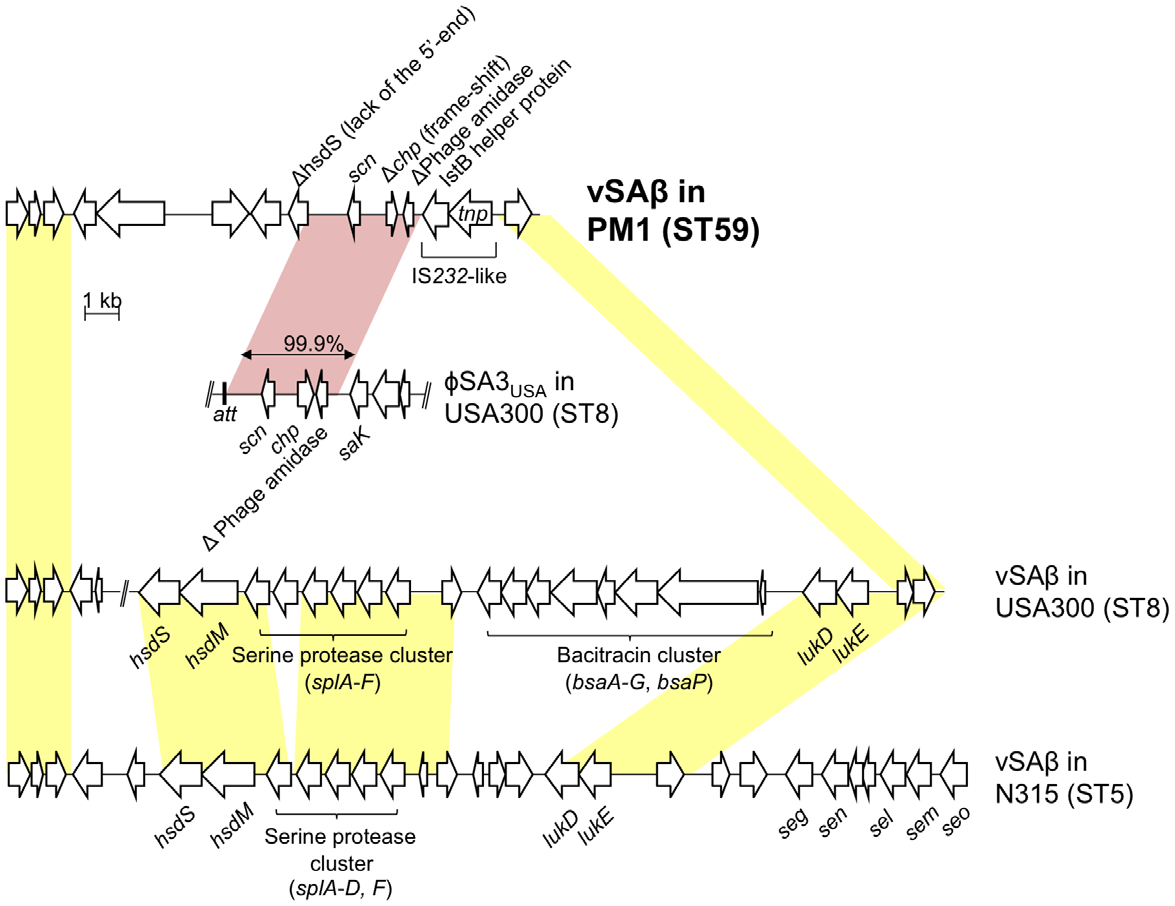
Structure of νSAβ in PM1. The 15,551-bp νSAβ structure in PM1 is shown in the upper part. This sequence was compared to the data of ST8 CA-MRSA strain USA300 FPR3757 (GenBank accession number NC_007793) and ST5 HA-MRSA strain N315 (GenBank accession number BA000018). PM1 regions homologous to φSA3_USA_ (of USA300) and νSAβs (of USA300 and N315) are shaded in pink and yellow, respectively. A Greek delta symbol indicates truncated coding sequence.

Genomic island vSAβ of MRSA strains, e.g. USA300, carried intact *hsdS* and *hsdM*, while vSAβ of PM1 carried truncated *hsdS* and lacked *hsdM* (Figure 5), although genomic island vSAα of PM1 carried an intact set of *hsdS* and *hsdM*, similar to USA300 (Figure 1). Lack of a set of *hsdS* and *hsdM* in PM1 may have contributed, in part, to PM1’s potential to acquire foreign DNA.

## Discussion

**Figure 6:**
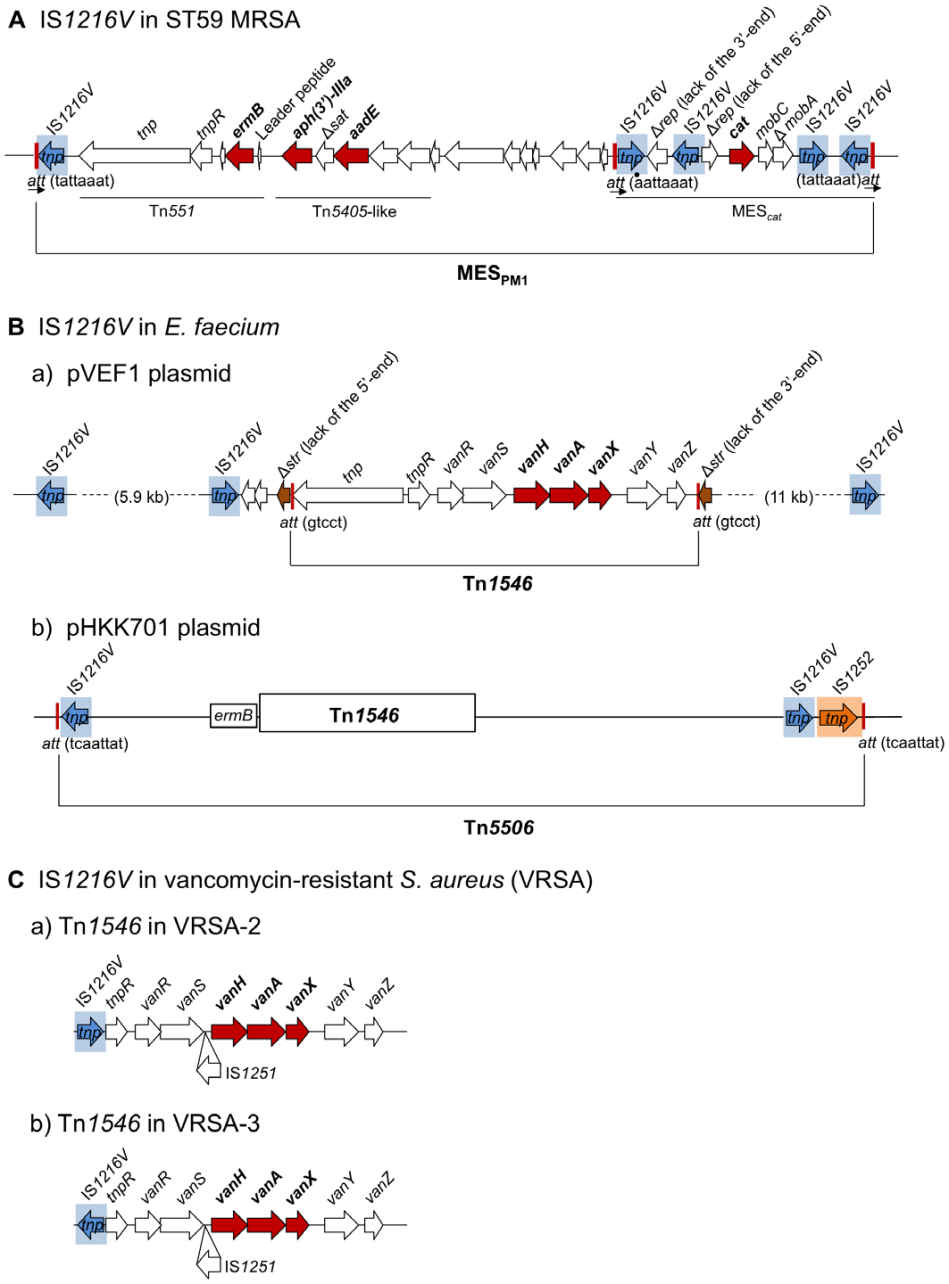
Comparison of IS*1216V*-containig mobile elements in MRSA and *E. faecium*. The structures of IS*1216V*-containing mobile elements: A, MES_PM1_ determined in this study; B, Tn*1546* and Tn*5506* in *E. faecium*; C, vancomycin resistance transposon Tn*1546* in VRSA. The IS*1216V* region with the transposase gene (*tnp*) is shaded in blue. The *att* site is indicated by a vertical red line. The data for pVEF1 are from GenBank (GenBank accession number AM296544). The data for pHKK701 and Tn*1546* in VRSA-2 and VRSA-3 were obtained from previously described maps [31], [32].

In this study, we performed comparative genomic analysis of a multidrug-resistant, PVL-positive ST59/SCC*mec*V CA-MRSA strain (PM1) and found MES_PM1_ on the PM1 chromosome, encoding resistance to erythromycin, clindamycin, kanamycin, streptomycin, and chloramphenicol. As shown in Figure 6A, MES_PM1_ exhibited typical features of a transposon, such as the presence of terminal repeats with 8-bp att duplications at both ends. The unique features of MES_PM1_ include the presence of IS*1216V* as a terminal direct repeat and a composite transposon region containing Tn*551* and Tn*5405*-like, both of which originate in enterococci (such as *E. faecium* and *E. faecalis*) [26], [33], [34], strongly suggesting the origin of MES_PM1_ in enterococci.

In *E. faecium*, IS*1216V* is considered to be part of a large mobile element containing Tn*1546* with *vanA* (encoding vancomycin resistance) [35], as shown in Figure 6B (a); however, a possible mobile structure with *att* duplication has not been reported. Moreover, in *E. faecium*, a large mobile element (Tn*5506*), which contains Tn*1546* and is flanked by hetero terminal sequences, IS*1216V* and IS*1252*, with 8-bp *att* duplications at both ends, has been described [32], as shown in Figure 6B (b); however, the *att* of Tn*5506* was different from the *att* of MES_PM1_ (Figure 6A and B [b]), and in the case of Tn*5506*, another copy of IS*1216V* was located adjacent to IS*1252* (Figure 6B [b]).

Transfer of the *van* genes (such as *vanA*) from enterococci to MRSA would be a great public health concern and threat, because vancomycin is the first-line agent for MRSA infections [3], [36], [37]. Unfortunately, Tn*1546* has already emerged in MRSA [27], as shown in Figure 6C; in this case, IS*1216V* is present near the *van* genes (although possible IS*1216V* repeats and *att* duplication have not been reported).

Therefore, this study demonstrates the first IS*1216V*-mediated mobile element structure with terminal direct repeats of IS*1216V* with *att* duplication at both ends. Interestingly, MES_PM1_ also contained another mobile element structure (MES*_cat_*) within the structure, which was flanked by inverted repeats of IS*1216V* with *att* duplication at both ends. Chloramphenicol resistance in MRSA is usually encoded by a plasmid [31], [38]; however, in this case, it was encoded by MES*_cat_* (as a part of MES_PM1_) on the chromosome. The chloramphenicol resistance region seemed to originate in *S. aureus*; therefore, MES_PM1_ is a highly composite structure with mobile elements originating in *Enterococcus* and *Staphylococcus* species.

This study clearly concludes that the acquisition mechanism of multidrug resistance in ST59 CA-MRSA in Taiwan is quite different from many other MRSA strains, but shows some similarity to VRSA cases, and that the MES_PM1_ acquisition event occurred most probably around 2007 or earlier in Taiwan, and then ST59 CA-MRSA carrying MES_PM1_ spread in Taiwan, followed by segregation events generating different resistance patterns.

The reason why ST59 CA-MRSA in Taiwan acquired MES_PM1_ is not known. The type I restriction-modification system, composed of HsdR (restriction), HsdM (modification), and HsdS (sequence specificity), is known to block horizontal gene transfer from other species into *S. aureus*, as well as to limit DNA exchange between the different lineages of *S. aureus* [39], [40]. Deficiency in the type I restriction-modification system would allow foreign DNA acquisition with high frequency. For example, a bovine pathogenic *S. aureus* strain, which is naturally mutated in both *hsdS* genes, has been reported displaying a “hyper-recipient” phenotype to acquire DNA from *Enterococcus* species [41]. In the case of ST59 CA-MRSA (strain PM1) in Taiwan, a copy of *hsdM* and *hsdS* in νSAβ was truncated, which might contribute to the acquisition of foreign DNA from enterococci such as IS*1216V* and MES_PM1_; further study is needed for elucidation.

In this study, a conjugative penicillinase plasmid (pPM1) in strain PM1 was successfully transferred by filter mating, while MES_PM1_ was not, suggesting that MES_PM1_ may not be a conjugative transposon.

In strain PM1, we also identified a novel mosaic penicillinase plasmid encoding tetracycline resistance (pPM1). The tetracycline resistance region (MES*_tet_*) could be a novel transposon flanked by IS*431*, which most probably originated in *S. aureus*. This structure also generated segregants (tetracycline-susceptible penicillinase plasmid), resulting in strains PM11 and PM18.

Regarding other genetic structures in ST59 CA-MRSA in Taiwan, we confirmed the sequence of SCC*mec*V of strain PM1 (formerly SCC*mec*VII) [24] and the sequence of PVL phage (φ5967PVL; φSA2_PM1_ in this study), which was originally reported with another strain (JCSC5967) by Zhang et al. [42]; they were 99.9% homologous.

During the course of this study, Huang et al. described the genome sequence of ST59 CA-MRSA strain M013 in Taiwan [43], although they did not describe the plasmid(s). According to their data, their strain (M013) is not multidrug-resistant; most probably it corresponds to strain PM18 in this study, a minor derivative of PM1 carrying only IS*1216V* at the MES_PM1_ insertion site and showing no resistance to non-β-lactam agents. M013 also does not carry φSA1_PM1_. In addition, three genes (*sasA*, *fnbA*, and *sdrC*) in M013 have more than 24-bp deletions or insertions. The comparison between the PM1 and M013 genomes is summarized in Table S1.

In conclusion, an IS*1216V*-mediated mobile element structure (MES_PM1_), encoding multidrug resistance and originating in enterococci, has emerged in PVL-positive ST59 CA-MRSA (e.g. strain PM1) in Taiwan. Although IS*1216V*, related to vancomycin resistance transposon Tn*1546*, has been reported in MRSA, MES_PM1_ represents the first mobile element flanked by IS*1216V* direct repeats with *att* duplication at both ends. MES_PM1_ also includes additional MES*_cat_* flanked by IS*1216V* inverted repeats with *att* duplication at both ends, although the *cat* region originated in *S. aureus*, indicating a highly composite formation in its evolution. The deficiency of a copy of *hsdM* and *hsdS* (in νSAβ) in strain PM1 might contribute to the acquisition of foreign DNA, such as IS*1216V* and MES_PM1_, from enterococci.

## Materials and Methods

### Bacterial Strains

Twenty-four PVL-positive ST59/SCC*mec*V CA-MRSA strains were collected from 2000 to 2006 in the Bacteriology Laboratory, National Taiwan University Hospital. These strains were isolated from patients with SSTI, bacteremia, respiratory tract infection, and surgical wounds [23]. USA1000 is an ST59 MRSA-type strain (of the SmaI PFGE type) isolated in the United States, and was kindly provided by L. K. McDougal and L.L. McDonald.

### Pyrosequence Analysis of the Genome

The PM1 genome was analyzed by pyrosequencing using a genome sequencer FLX system (Roche Diagnostics, Branford, CT, USA). It yielded 93 Mb raw sequences by 236,943 reads, corresponding to approximately 33.2-fold of the genome size. Contigs were then assembled according to the two genomes of ST8 MRSA USA300 FPR3757 (GenBank accession number NC_007793) and ST398 MRSA human strain S0385 (GenBank accession number AM990992). Open reading frames were searched for using the software in silico MolecularCloning (version 4.2) (In Silico Biology, Yokohama, Japan) or DNAman software package (Version 6) (Lynnon Biosoft, Quebec, Canada).

### Entire Sequencing of Mobile Elements and a Plasmid

The gaps between contigs, related to mobile elements, were filled by PCR and sequencing. We also assembled contigs using an LA PCR in vitro cloning kit (Takara Bio, Otsu, Japan) according to the manufacturer’s instructions. In brief, after digestion with suitable restriction enzymes and ligation with the corresponding cassette adapters, amplification was performed with cassette primers and target-specific primers.

### PCR Analysis of the MES_PM1_ Structure in Other PVL-positive ST59 CA-MRSA Strains

Based on the determined entire sequence of MES_PM1_, a series of primer sets was designed (Table 3) and used to screen the genetic constitution of MES_PM1_. In PCR reactions, 10% glycerol or 4% DMSO was added in order to dissolve the secondary structure of IS*1216V*.

**Table 3:** Primers used for PCR mapping of the MES_PM1_ locus.

Primer name	Sequence (5′ to 3′)
11F2	AGGCGAAACATTGGAAGT
ermBR	GCAATGAAACACGCC
ermB	AGTAACGGTACTTAAATTGTTTAC
aadE	ACTGGCTTAATCAATTTGGG
1F	AGTAGCCTTTCCCTCACTT
35R	GCTTTGACGCTATGACGA
35F	CCTTACCAGTTGTTCCGAA
LA-R2	CCCATGCAGGTTTCAAAATGTGTAAGTCA
LA-F1	ACACCAGAACAATGTAATCAACTAGGGT
23R	TGTTCCAAAGAGCCATCA
23F	CATCAGGAGGGCAAGAA
48R3	TCAGGCGTGAGAATAAATGA
catR	GCATTTGATTCTGTCCA

### Susceptibility Testing

Antimicrobial susceptibility tests were performed by the agar dilution method with Hueller-Hinton agar (Difco, Sparks, MD, USA) according to the guidelines of the Clinical and Laboratory Standards Institute [44].

### Conjugative Transfer

Donor strains were mated with *S. aureus* RN2677 (recipient strain, which is restriction-negative and resistant to rifampicin and novobiocin) on membrane filters on tryptic soy agar (Difco) (filter mating), as previously described [45]; RN2677 was used as a recipient because it carried no plasmids and had non-transmissible drug resistance (recipient) markers. The resistance genes of transconjugants were examined by PCR as previously described [23].

### Plasmid Analysis

Plasmid DNA was isolated using a plasmid midi kit (Qiagen, Hilden, Germany) and lysostaphin (Wako Pure Chemicals, Osaka, Japan), following the manufacturer’s instructions.

## Supporting Information

Table S1 Comparative genomic analysis between PM1 and M013.
(XLS)
